# The *HLA-B* –21 M/T dimorphism associates with disease severity in COVID-19

**DOI:** 10.1038/s41435-024-00302-6

**Published:** 2024-11-01

**Authors:** Benedikt Strunz, Pouria Momayyezi, Eleni Bilev, Jagadeeswara Rao Muvva, Puran Chen, Jonna Bister, Marie Schaffer, Mira Akber, Martin Cornillet, Karolinska KI/K COVID-19 Study Group, Benedikt Strunz, Jagadeeswara Rao Muvva, Puran Chen, Mira Akber, Martin Cornillet, Soo Aleman, Lena Berglin, Helena Bergsten, Susanna Brighenti, Demi Brownlie, Marcus Buggert, Marta Butrym, Benedict J. Chambers, Angelica Cuapio, Isabel Diaz Lozano, Lena Dillner, Therese Djärv, Majda Dzidic, Johanna Emgård, Lars I. Eriksson, Malin Flodström-Tullberg, Hedvig Glans, Jean-Baptiste Gorin, Jonathan Grip, Alvaro Haroun-Izquierdo, Elisabeth Henriksson, Laura Hertwig, Sadaf Kalsum, Tobias Kammann, Jonas Klingström, Efthymia Kokkinou, Egle Kvedaraite, Marco Giulio Loreti, Magdalini Lourda, Kimia T. Maleki, Karl-Johan Malmberg, Nicole Marquardt, Johan Mårtensson, Christopher Maucourant, Jakob Michaëlsson, Jenny Mjösberg, Kirsten Moll, Pontus Nauclér, Anna Norrby-Teglund, Laura M. Palma Medina, Tiphaine Parrot, Andre Perez-Potti, Björn P. Persson, Lena Radler, Dorota Religa, Emma Ringqvist, Olga Rivera-Ballesteros, Olav Rooyackers, Johan K. Sandberg, John Tyler Sandberg, Takuya Sekine, Ebba Sohlberg, Tea Soini, Anders Sönnerborg, Kristoffer Strålin, Mattias Svensson, Janne Tynell, Christian Unge, Renata Varnaite, Andreas von Kries, David Wullimann, Hans-Gustaf Ljunggren, Niklas K. Björkström, Quirin Hammer, Amir Horowitz, Karl-Johan Malmberg, Olav Rooyackers, Soo Aleman, Hans-Gustaf Ljunggren, Niklas K. Björkström, Kristoffer Strålin, Quirin Hammer

**Affiliations:** 1 Center for Infectious Medicine, Department of Medicine Huddinge, Karolinska Institutet, Karolinska University Hospital, Stockholm, Sweden; 2 Department of Oncological Sciences, Precision Immunology Institute, Tisch Cancer Institute, Icahn School of Medicine at Mount Sinai, New York, NY USA; 3 Department of Cancer Immunology, Institute for Cancer Research, Oslo University Hospital and University of Oslo, Oslo, Norway; 4 Division of Anesthesiology and Intensive Care, Department of Clinical Science, Intervention and Technology, Karolinska Institutet, Stockholm, Sweden; 5 Department of Infectious Diseases, Karolinska University Hospital, Stockholm, Sweden; 6 Division of Infectious Diseases and Dermatology, Department of Medicine Huddinge, Karolinska Institutet, Stockholm, Sweden; 7 Department of Emergency Medicine, Karolinska University Hospital, Stockholm, Sweden; 8 Department of Perioperative Medicine and Intensive Care, Karolinska University Hospital, Stockholm, Sweden; 9 Theme Aging, Karolinska University Hospital, Stockholm, Sweden

**Keywords:** Disease genetics, Immunogenetics, Innate lymphoid cells

## Abstract

Host genetics shape immune responses and influence severity of infectious diseases. The *HLA-B* –21 M/T dimorphism tunes the functionality of natural killer (NK) cells expressing the inhibitory receptor NKG2A. NKG2A^+^ NK cells have been reported to recognize SARS-CoV-2-infected cells, but it remains unclear whether the *HLA-B* –21 M/T dimorphism associates with COVID-19 severity. Here, we investigated the influence of the *HLA-B* –21 M/T dimorphism in a cohort of 230 unvaccinated patients hospitalized with COVID-19 and requiring respiratory support. We found that *HLA-B* –21 M/M genotypes were more prevalent in patients with moderate compared to severe COVID-19 (6.0% vs. 0.9%). Comparison of age- and sex-matched sub-groups revealed that patients with M/M genotypes required mechanical respiratory support less frequently (OR = 0.13, 95% CI = 0.01-0.76, *P* = 0.013). Furthermore, patients with M/M genotypes showed a coordinately shifted signature of clinical laboratory parameters, coinciding with elevated serum levels of the anti-viral cytokine IFN-γ. These findings demonstrate that *HLA-B* variants associate with COVID-19 severity and suggest that the robust functionality of NKG2A^+^ NK cells in patients carrying the M/M genotype may contribute to protection from severe disease.

## Introduction

Host genetics contribute to divergent disease courses in patients who develop coronavirus disease 2019 (COVID-19) following infection by severe acute respiratory syndrome coronavirus 2 (SARS-CoV-2). Numerous genetic loci associated with COVID-19 have been identified, including variants that increase susceptibility as well as those that protect against severe disease [1].

Natural killer (NK) cells are innate immune cells that respond early during acute viral infections. In line with their anti-viral functions, high numbers of peripheral NK cells have been reported to correlate with rapid clearance of SARS-CoV-2 [2]. NK cells in the peripheral blood of patients with severe COVID-19 were shown to be reduced in numbers, strongly activated, and partially dysfunctional [3]. Alternatively, NK cells could contribute to COVID-19 pathology, for instance by attacking uninfected bystander cells [4].

The functionality of NK cells is in part regulated by genetic factors, with variation in the *HLA-B* gene governing the functional capacity of NK cells expressing the receptor NKG2A. A dimorphism at position –21 of HLA-B (SNV rs1050458) encodes for either threonine (T) or methionine (M), generating leader peptides with lower (T) or higher (M) presentation on HLA-E [5]. Since presentation of these leader peptides at steady state fine-tunes the effector function of NKG2A^+^ NK cells, the *HLA-B* –21 dimorphism thus separates individuals into groups with genetically hard-wired highest functional potency (M/M) and with comparably reduced functional potency (M/T and especially T/T) [6]. This dimorphism has been implicated in controlling NK cell functions in anti-cancer as well as in anti-viral responses [7, 8]. However, whether the *HLA-B* –21 dimorphism affects the risk of developing severe COVID-19 remains unclear.

Here, we show that rare *HLA-B* –21 M/M genotypes are enriched in patients with moderate COVID-19 and depleted in patients with severe manifestations. In age- and sex-matched patient sub-groups, we uncover a coordinated shift in a multiparametric disease signature, further indicating reduced severity in M/M patients. Moreover, plasma protein profiling revealed elevated levels of the anti-viral cytokine interferon-gamma (IFN-γ) in patients with M/M genotypes. Together, these data show that genetic variations in *HLA-B*, which tune NK cell functionality, are associated with COVID-19 severity.

## Materials and methods

The study was approved by the Swedish Ethical Review Authority (DNR 2020-01558) and included *n* = 230 unvaccinated patients with COVID-19 who required respiratory support at the Karolinska University Hospital in Stockholm, Sweden. All patients were confirmed positive for SARS-CoV-2 by polymerase chain reaction, unrelated, and recruited during 2020, before implementation of COVID-19 vaccination. Of the complete cohort, *n* = 102 patients received low-flow oxygen therapy, *n* = 30 high-flow nasal oxygen, *n* = 10 non-invasive mechanical ventilation, *n* = 82 invasive mechanical ventilation, and *n* = 6 were treated with extracorporeal membrane oxygenation (ECMO). Severe COVID-19 was defined by either requiring non-invasive mechanical ventilation, invasive mechanical ventilation, or ECMO or by having deceased during treatment (*n* = 114); all other patients were classified as moderate disease (*n* = 116). All patients were sampled during their hospital stay for whole blood and serum, which was frozen at −20 °C or −80 °C, respectively, until analysis.

*HLA-B* typing was performed by bead-based reverse sequence-specific oligonucleotides (rSSO) using LABType SSO Class I B Locus Typing Test kit and LABScan 100 (both ThermoFisher).

Additional information on the cohort, materials, and methods are available in the Supplementary information.

## Results

**Fig. 1:**
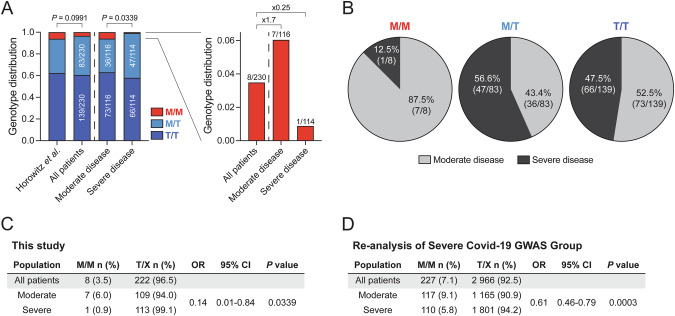
The *HLA-B* –21 M/M genotype is differentially distributed across COVID-19 severity. **A** Distribution of M/M, M/T, and T/T genotypes in a reference (*n* = 8192) [6], all patients included in the study cohort (*n* = 230), patients with moderate disease (*n* = 116), and patients with severe disease (n = 114). Right bar graphs display M/M genotypes and fold enrichment/depletion compared to all patients. **B** Distribution of disease manifestation in patients with M/M (*n* = 8), M/T (*n* = 83), and T/T genotypes (*n* = 139). **C** Tabular summary of the study cohort (*n* = 230). **D** Tabular summary of a cohort previously published by Ellinghaus and colleagues [9] (*n* = 3193). Two-tailed Chi-square test (**A**, **C**, **D**).

To study the influence of *HLA-B* variants on COVID-19 severity, we determined *HLA-B* alleles in 230 unvaccinated patients who were hospitalized with COVID-19 and required respiratory support during the early stages of the pandemic (Figure S1A, Table 1S). Next, we imputed the *HLA-B* –21 M/T dimorphism (SNV rs1050458; Table S2) and assembled M/M, M/T, and T/T genotypes. Assessing genotype distribution revealed that 3.5% of patients were of M/M, 36.1% of M/T, and 60.4% of T/T genotypes, aligning with previously reported frequencies [6]. When classifying hospitalized patients into moderate and severe COVID-19, we observed that M/M genotypes were more prevalent in patients with moderate disease (6.0%, 1.7-fold higher) and were proportionally depleted from the group with severe COVID-19 (0.9%, 4-fold lower; Fig. 1A). Accordingly, only few patients with M/M genotypes presented with severe disease (12.5%), while this frequency was similarly increased among patients with M/T and T/T genotypes (56.6% and 47.5%; Fig. 1B), providing a rationale for grouping of these two genotypes into one T/X group. The observed genotype distributions among the severity groups resulted in a significant association of M/M genotypes with protection from severe disease in the present cohort (OR = 0.14, 95% CI = 0.01-0.84, *P* = 0.0339; Fig. 1C). This association was also detected in a re-analysis of a previously published dataset [9] (OR = 0.61, 95% CI = 0.46-0.79, *P* = 0.0003; Fig. 1D).

**Fig. 2:**
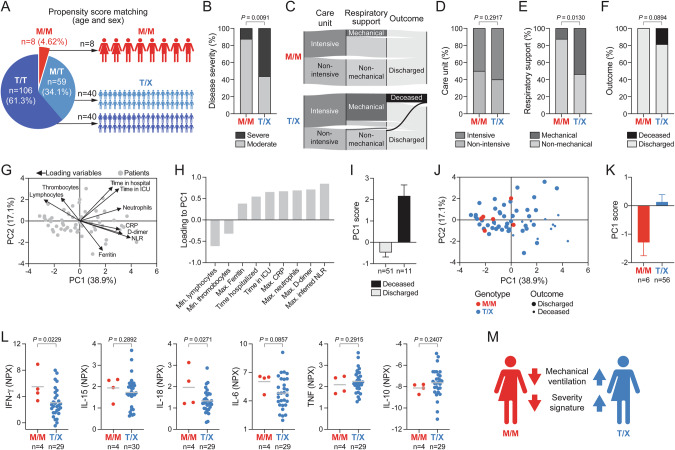
Patients with M/M genotypes require less mechanical respiratory support and display a signature of reduced severity in an age- and sex-matched sub-cohort. **A** Schematic illustration of propensity score matching for age and sex. **B** Distribution of disease manifestations in genotype groups. **C** Disease courses of genotype groups. Distribution of patients **D** requiring intensive or non-intensive care, **E** requiring mechanical or non-mechanical respiratory support, and **F** with deceased or discharged outcome. PCA of clinical laboratory parameters. **G** Biplot showing individual patients (grey dots) and loading of variables (arrows). **H** Contribution of parameters to loading to PC1. **I** PC1 scores of discharged and deceased patients. Bars indicate mean and error bars SEM. **J** Score plot of patients stratified for genotype group and outcome. **K** PC1 scores of M/M and T/X genotype groups. Bars indicate mean and error bars SEM. **L** Serum levels of selected cytokines determined by proximity extension assay. Dots represent individual patients and lines indicate mean. Numbers below graphs denote number of included samples. **M** Graphical summary of findings. *n* = 8 for M/M, *n* = 80 for T/X (**B**–**F**), *n* = 6 for M/M, *n* = 56 for T/X (**G**–**K**), and *n* = 4 for M/M, *n* = 27 for T/X (**L**). One-tailed chi-squared test (**B**, **D**–**F**) and one-tailed t-test (**L**).

We next asked whether the presence of M/M genotypes represented an independent protective factor within the present cohort. To address this, we performed propensity score matching for age and sex, selecting a sub-cohort of eight patients with M/M genotypes and 80 patients with T/X genotypes (Fig. 2A) with comparable Charlson Comorbidity Index and similar body mass index (BMI; Fig. S1B; Table S3). In this matched sub-cohort, patients with M/M genotypes also presented less frequently with severe disease (12.5% vs. 56.3%; OR = 0.11, 95% CI = 0.01–0.69, *P* = 0.009; Fig. 2B), suggesting that M/M genotypes confer protection independent of confounding variables such as age and sex. Disease course analyses revealed that M/M patients showed a significantly reduced requirement for mechanical ventilation (12.5% vs. 53.8%; OR = 0.13, 95% CI = 0.01–0.76, *P* = 0.013; Fig. 2C–E). Moreover, all M/M patients were discharged, while 18.8% of patients with T/X genotypes deceased during treatment, although this observation did not reach statistical significance (*P* = 0.089; Fig. 2F).

We further investigated clinical laboratory parameters indicative of COVID-19 severity. Patients with M/M genotypes exhibited favorable clinical parameters such as less pronounced lymphopenia and lower neutrophil-to-lymphocyte-ratio, suggesting ameliorated disease profiles despite interindividual heterogeneity (Fig. S2). To explore the disease spectrum in-depth, we integrated all clinical laboratory indicators into a multidimensional dataset containing 558 datapoints of 62 patients. Dimensionality reduction by principal component analysis (PCA) uncovered that individual patients were projected mainly along principal component 1 (PC1; Fig. 2G). PC1 was negatively associated with lymphocyte and thrombocyte counts, while it was positively associated with disease markers such as ferritin, time hospitalized and in intensive care, CRP, maximal neutrophil counts, D-dimer, and neutrophil-to-lymphocyte-ratio (Fig. 2H), implying that low scores in PC1 represent moderate disease whereas high scores point towards severe COVID-19. In agreement with this, patients who were discharged displayed negative average PC1 scores, whereas patients who deceased showed positive scores (Fig. 2I). Importantly, patients carrying M/M genotypes were largely confined to negative scores in PC1 (mean −1.30; range −2.33 to 0.18), while patients with T/X genotypes were diffusely distributed along PC1 (mean 0.14; range −3.14 to 4.42; Fig. 2J, K).

Finally, we assessed selected plasma cytokines related to anti-viral activity and systemic immune activation in a limited number of patients. Cytokine profiling using proximity extension assay implied elevated levels of the anti-viral cytokine IFN-γ in M/M patients (Fig. 2L), which was accompanied by increased quantities of the NK cell-stimulating cytokine IL-18, whereas other cytokines indicative of systematic immune activation such as IL-6, TNF or IL-10 were similar between M/M and T/X patients (Fig. 2L).

Collectively, these results demonstrate that *HLA-B* –21 M/M genotypes associate with protection from severe manifestations of COVID-19 (Fig. 2M).

## Discussion

The role of NK cells in limiting COVID-19 severity remains incompletely understood. In this study, we provide evidence that patients with *HLA-B* –21 M/M genotypes follow less severe COVID-19 disease trajectories. This is in agreement with previous findings demonstrating enhanced functionality of NKG2A^+^ NK cells in M/M donors [6] and the response of NKG2A^+^ NK cells against SARS-CoV-2-infected cells [10].

Variation in HLA and its association with COVID-19 severity or outcome has been investigated in numerous studies, mainly focusing on HLA-mediated presentation of viral antigens to T cells [11]. For instance, HLA-B*15:01 is associated with asymptomatic SARS-CoV-2 infection due to pre-existing T-cell immunity against an HLA-B*15:01-restricted viral peptide [12]. In contrast, studies on the contribution of HLA molecules to regulate the effector capacity of NK cells through the process of NK cell education have remained scarce. Here, we provide evidence that HLA-B variants associate with COVID-19 severity based on the *HLA-B* –21 M/T dimorphism. Thus, our findings point towards a mechanism by which host resistance to SARS-CoV-2 may be genetically determined by HLA alleles indirectly, via functional tuning of NKG2A-expressing NK cells.

The indirect regulation of NK cell functionality by the *HLA-B* –21 M/T dimorphism is well described. Due to the supply of leader peptides with lower (T) or higher (M) presentation on HLA-E [5], NK cells expressing the HLA-E-binding inhibitory receptor NKG2A show a higher degree of education in the presence of M compared to T alleles [6]. Consequently, the presence of at least one M allele correlates with pronounced functional responses of NKG2A^+^ NK cells [6, 8]. We have recently described that the non-structural protein 13 (Nsp13) of SARS-CoV-2 contains an HLA-E-restricted peptide [10]. When presented on HLA-E, the Nsp13_232-240_ peptide hinders binding to the inhibitory receptor NKG2A, reducing inhibition and thereby promoting effector functions of NKG2A-expressing NK cells [10]. We further detected unleashed activity of NKG2A^+^ NK cells ex vivo from patients and suppression of SARS-CoV-2 replication by NKG2A^+^ NK cells in vitro [10]. Therefore, it is conceivable that protective effects of M/M genotypes in COVID-19 are similarly exerted through enhanced NKG2A^+^ NK cell responses against SARS-CoV-2-infected cells. The observation that milder disease manifestations in patients carrying M/M genotypes coincided with heightened levels of the anti-viral cytokine IFN-γ potentially suggests that secretion of IFN-γ by NK cells could be one mechanism contributing to protection.

Apart from variation in HLA-B, other factors such as the expression level of HLA-A govern NKG2A^+^ NK cell functional capacity [13]. In addition to the HLA-B -> HLA-E -> NKG2A axis, COVID-19 severity appears to be influenced by genetic variation in other NK cell receptors. Deletion of *KLRC2* (encoding NKG2C) represents a risk factor for severe disease [14], whereas the presence of KIR2DS4*004 associates with severity [15], together supporting the role of NK cells and their receptors in combating SARS-CoV-2.

Previous studies have reported associations of *HLA-B* alleles with COVID-19. HLA-B*44 was associated with the incidence of COVID-19 [16] and HLA-B*35 was enriched in COVID-19 patients compared to controls [17], while HLA-B*40 and HLA-B*55 were enriched, but not statistically significant [18]. Since these four *HLA-B* alleles encode for a T at position –21, it is tempting to speculate that such observations could in part be due to a lower functionality of NKG2A^+^ NK cells. Conversely, HLA-B*15 encodes for a T but correlates with asymptomatic infection [12], highlighting that comprehensive future studies are required to fully delineate these associations and dissect their impact on NK cells or pre-existing T cell responses. Moreover, although all three *HLA-B* –21 genotypes were reported to be present in all human populations [6], their relative frequencies vary considerably and the highest prevalence of M/M genotypes is found in Europe [6], which may influence results due to underlying population structures and generate an additional layer of complexity.

In conclusion, we demonstrate that the –21 M/T dimorphism of the *HLA-B* gene associates with COVID-19 severity, suggesting that a genetically hard-wired potent functionality of NKG2A^+^ NK cells in M/M patients may contribute to protection against severe illness in a cohort of unvaccinated patients receiving respiratory support.

## Supplementary information

Supplemental material (combined in one .pdf file)

## Data Availability

In addition to the data and methods reported in the article and the Supplementary information, data are available upon request pending data transfer agreements approved by local authorities.
