# Supplementary material for: The HLA-B –21 M/T dimorphism associates with disease severity in COVID-19
Source: Genes Immun. 2024 Nov 1;26(1):70–4. doi: 10.1038/s41435-024-00302-6 (PMC11832411; doi:10.1038/s41435-024-00302-6)
Supplement: Supplementary file 1 — Supplemental material (combined in one .pdf file) [file 41435_2024_302_MOESM1_ESM.pdf]

**The *HLA-B* –21 M/T dimorphism associates with disease severity in COVID-19**

Benedikt Strunz*, Pouria Momayyezi*, Eleni Bilev, Jagadeeswara Rao Muvva, Puran Chen, Jonna Bister, Marie Schaffer, Mira Akber, Martin Cornillet, Karolinska KI/K COVID-19 Study Group, Amir Horowitz, Karl-Johan Malmberg, Olav Rooyackers, Soo Aleman, Hans-Gustaf Ljunggren, Niklas K Björkström, Kristoffer Strålin and Quirin Hammer.

**Supplementary information**

## Supplementary figures

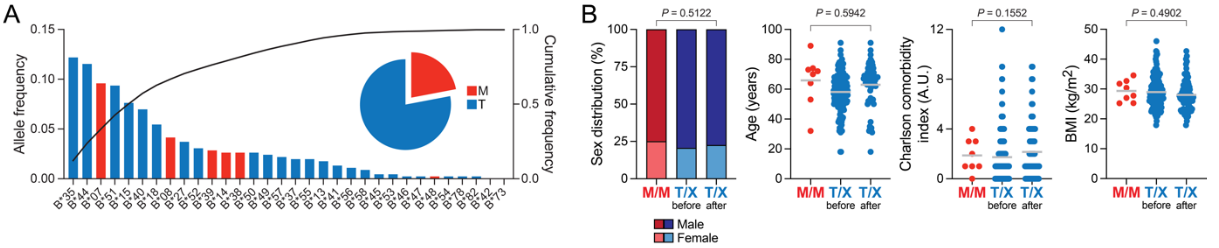


**Figure S1. Allele frequencies and matching comparison.** (**A**) Frequency of HLA-B alleles in the study cohort (n=230) obtained class I B locus typing. Blue and red bars indicate *HLA-B* –21 T and M, respectively. Pie chart shows allele distribution. (**B**) Comparison of M/M and T/X cohorts in sex, age, Charlson comorbidity index, and body mass index (BMI) before and after propensity-score matching. Dots represent individual patients and lines indicate mean (n=8 for M/M, n=165 for T/X before, and n=80 for T/X after matching). Two-tailed chi-squared test and two-tailed t-test (B).

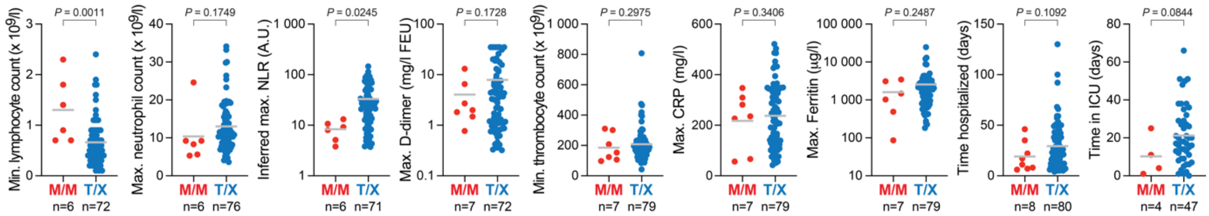


**Figure S2. Clinical laboratory parameters.**

Clinical laboratory parameters. Dots represent individual patients and lines indicate mean. Numbers below graphs denote number of included samples. One-tailed t-test.

## Supplementary tables

### Table S1. Summarized characteristics of complete cohort.

| | M/M | M/T | T/T |
|---|---|---|---|
| **Group size**, n | 8 | 83 | 139 |
| **Age**, mean [range] years | 66 [32-89] | 63 [18-99] | 58 [24-89] |
| **Sex**, % (female/male) | 25.0/75.0 | 24.1/75.9 | 21.6/78.4 |
| **Body mass index**, mean [range] kg/m$^2$ | 29.3 [25.2-34.6] | 27.7 [17.7-42.7] | 28.6 [20.6-46.0][a] |
| **Charlson comorbidity index**, mean [range] | 1.9 [0-4] | 2.1 [0-9] | 1.6 [0-12] |
| **Severity**, % (moderate/severe) | 87.5/12.5 | 43.4/56.6 | 52.5/47.5 |
| **Care unit**, % (non-intensive/intensive) | 50.0/50.0 | 50.6/49.4 | 49.6/50.4 |
| **Respiratory support**[b], % (non-mechanical/mechanical) | 87.5/12.5 | 43.4/56.6 | 43.9/56.1 |
| **Outcome**, % (discharged/deceased) | 100.0/0.0 | 79.5/20.5 | 87.1/12.9 |

[a] missing value for one patient

[d] non-mechanical defined as low-flow oxygen therapy or high-flow nasal cannula; mechanical defined as non-invasive mechanical ventilation, invasive mechanical ventilation, or ECMO.

**Table S2.** ***HLA-B*** **–21 M/T reference across HLA-B allele groups.**

| Allele group | *HLA-B* –21 M/T dimorphism (SNV rs1050458) |
|---|---|
| HLA-B*07 | M |
| HLA-B*08 | M |
| HLA-B*13 | T |
| HLA-B*14 | M |
| HLA-B*15 | T |
| HLA-B*18 | T |
| HLA-B*27 | T |
| HLA-B*35 | T |
| HLA-B*37 | T |
| HLA-B*38 | M |
| HLA-B*39 | M |
| HLA-B*40 | T |
| HLA-B*41 | T |
| HLA-B*42 | M |
| HLA-B*44 | T |
| HLA-B*45 | T |
| HLA-B*46 | T |
| HLA-B*47 | T |
| HLA-B*48 | M |
| HLA-B*49 | T |
| HLA-B*50 | T |
| HLA-B*51 | T |
| HLA-B*52 | T |
| HLA-B*53 | T |
| HLA-B*54 | T |
| HLA-B*55 | T |
| HLA-B*56 | T |
| HLA-B*57 | T |
| HLA-B*58 | T |
| HLA-B*59 | T |
| HLA-B*67 | M |
| HLA-B*73 | M |
| HLA-B*78 | T |
| HLA-B*81 | M |
| HLA-B*82 | T |
| HLA-B*83 | T |

**Table S3. Summarized characteristics of the cohort after propensity-score matching.**

| | M/M | T/X |
|---|---|---|
| **Group size**, n | 8 | 80 |
| **Age**, mean [range] years | 66 [32-89] | 63 [18-91] |
| **Sex**, % (female/male) | 25.0/75.0 | 22.5/77.5 |
| **Body mass index**, mean [range] kg/m$^2$ | 29.3 (25.2-34.6) | 28.0 (17.9-42.7) |
| **Charlson comorbidity index**, mean [range] | 1.9 [0-4] | 2.2 [0-9] |
| **Severity**, % (moderate/severe) | 87.5/12.5 | 43.7/56.3 |
| **Care unit**, % (non-intensive/intensive) | 50.0/50.0 | 40.0/60.0 |
| **Respiratory support**, % (non-mechanical/mechanical) | 87.5/12.5 | 46.3/53.7 |
| **Outcome**, % (discharged/deceased) | 100.0/0.0 | 81.3/18.7 |

## Supplementary materials and methods

### Study cohort

As approved by the Swedish Ethical Review Authority (DNR 2020-01558), patients hospitalized with acute COVID-19 at the Karolinska University Hospital in 2020, before the introduction of COVID-19 vaccination, were included in the study. We selected n=230 unrelated patients requiring respiratory support for *HLA-B* typing using RNeasy DNeasy Blood & Tissue Kit (Qiagen) for DNA isolation and LABType SSO Class I B Locus Typing Test kit as well as LABScan 100 (both ThermoFisher) for typing. The *HLA-B* –21 M/T dimorphism (SNV rs1050458) was imputed as described previously[1, 2] by assigning the dimorphism based on major allele groups as listed in Table S2 and without taking rare subtypes into account. Additional information about the complete cohort is available in Table S1.

From the cohort of n=230 patients, we had additional clinical laboratory parameters available for n=173 patients. For this sub-group of patients, the additional parameters included minimal lymphocyte count during hospital stay, maximal neutrophil count, minimal thrombocyte count, maximal ferritin concentration, maximal C-reactive protein (CRP) levels, maximal D-dimer levels, and length of time of hospital stay as well as length of time on intensive care unit. We inferred a maximal neutrophil-lymphocyte-ratio (NLR) by dividing the maximal neutrophil count by the minimal lymphocyte count. Within this sub-group of n=173 patients with additional data, we performed propensity-score matching for age and sex in a 1:5 ratio for M/M:M/T and for M/M:T/T using the MatchIt package (https://kosukeimai.github.io/MatchIt/)[3] in R 4.1.3, resulting in a matched T/X group (n = 80). A comparison of age, sex, Charlson comorbidity index, and body mass index (BMI) before and after matching is available in Figure S1B, an overview of patient characteristics is available in Table S3.

**Re-analysis of published dataset**

Details on study design and patient groups have been previously described[4]. In brief, the 3193 COVID-19 cases reported by Ellinghaus and colleagues were stratified into moderate disease (oxygen therapy) or severe disease (mechanical ventilation; either non-invasive, invasive, or ECMO), the *HLA-B* –21 dimorphism was imputed as described above, and the association between M/M genotypes and disease severity was tested on summary data. Additional information on the cohort is available in the original publication[4].

**Principal component analysis**

Principal component analysis (PCA) was performed in GraphPad Prism to reduce the dimensionality of the multivariate clinical laboratory dataset comprising nine variables for $N =$ 62 patients. In brief, the data were standardized to a mean of 0 and an SD of 1, and PC1 and PC2 were selected based on their proportion of covered variance (38.9% and 17.1%, respectively).

**Cytokine profiling**

Serum cytokine profiling was performed by proximity extension assay. In brief, serum samples were analyzed with the Olink Explore platform as described elsewhere[5] and the analysis was focused on selected analytes including cytokines related to anti-viral function of NK cells (IFN-γ as effector cytokine and IL-18 as well as IL-15 as activators of NK cells) as well as on cytokines associated with systemic immune activation (IL-6, TNF, and IL-10). To avoid bias due to treatment regimens, patients treated with corticosteroids or cytokine blockade prior to sampling were excluded from the analysis. Additionally, samples and assays failing quality control were excluded and outliers were identified by Grubbs' test using an alpha of

0.05 and subsequently removed from further analyses. Data are displayed as normalized protein expression (NPX), a $\log_2$-scaled arbitrary unit used for relative quantification. Additional information and data on soluble factor profiling are available from the corresponding author upon request pending data transfer agreements.

**Statistical analyses**

Two-tailed chi-squared test was used for comparing genotype frequency distributions and to explore associations between genotypes and disease severity. Single hypotheses were tested by using one-tailed chi-squared or unpaired t-tests, for instance to analyze clinical laboratory parameters and cytokine levels between M/M and T/X genotype groups. Statistical tests were performed in GraphPad Prism, and all performed tests as well as all obtained *P* values are indicated in the figures or figure legends.

**Supplementary references**


1. Horowitz A, Djaoud Z, Nemat-Gorgani N, Blokhuis J, Hilton HG, Beziat V *et al.* Class I HLA haplotypes form two schools that educate NK cells in different ways. *Sci Immunol* 2016; **1**(3).
2. Petersdorf EW, Carrington M, O'HUigin C, Bengtsson M, De Santis D, Dubois V *et al.* Role of HLA-B exon 1 in graft-versus-host disease after unrelated haemopoietic cell transplantation: a retrospective cohort study. *Lancet Haematol* 2020; **7**(1)**:** e50-e60.
3. Ho D, Imai K, King G, Stuart EA. MatchIt: Nonparametric Preprocessing for Parametric Causal Inference. *Journal of Statistical Software* 2011; **42**(8)**:** 1 - 28.
4. Severe Covid GG, Ellinghaus D, Degenhardt F, Bujanda L, Buti M, Albillos A *et al.* Genomewide Association Study of Severe Covid-19 with Respiratory Failure. *The New England journal of medicine* 2020; **383**(16)**:** 1522-1534.
5. Strunz B, Maucourant C, Mehta A, Wan H, Du L, Sun D *et al.* Type I Interferon Autoantibodies Correlate With Cellular Immune Alterations in Severe COVID-19. *The Journal of infectious diseases* 2024.
